# Classification of Greek Olive Oils from Different Regions by Machine Learning-Aided Laser-Induced Breakdown Spectroscopy and Absorption Spectroscopy

**DOI:** 10.3390/molecules26051241

**Published:** 2021-02-25

**Authors:** Nikolaos Gyftokostas, Eleni Nanou, Dimitrios Stefas, Vasileios Kokkinos, Christos Bouras, Stelios Couris

**Affiliations:** 1 Department of Physics, University of Patras, 26504 Patras, Greece; n.guftokostas@iceht.forth.gr (N.G.); e.nanou@iceht.forth.gr (E.N.); d.stefas@iceht.forth.gr (D.S.); 2 Institute of Chemical Engineering Sciences (ICE-HT), Foundation for Research and Technology-Hellas (FORTH), 26504 Patras, Greece; 3 Department of Computer Engineering & Informatics, University of Patras, 26504 Patras, Greece; kokkinos@cti.gr (V.K.); bouras@cti.gr (C.B.)

**Keywords:** LIBS, laser-induced breakdown spectroscopy, visible absorption, machine learning, olive oil, classification, PCA, LDA, SVC

## Abstract

In the present work, the emission and the absorption spectra of numerous Greek olive oil samples and mixtures of them, obtained by two spectroscopic techniques, namely Laser-Induced Breakdown Spectroscopy (LIBS) and Absorption Spectroscopy, and aided by machine learning algorithms, were employed for the discrimination/classification of olive oils regarding their geographical origin. Both emission and absorption spectra were initially preprocessed by means of Principal Component Analysis (PCA) and were subsequently used for the construction of predictive models, employing Linear Discriminant Analysis (LDA) and Support Vector Machines (SVM). All data analysis methodologies were validated by both “k-fold” cross-validation and external validation methods. In all cases, very high classification accuracies were found, up to 100%. The present results demonstrate the advantages of machine learning implementation for improving the capabilities of these spectroscopic techniques as tools for efficient olive oil quality monitoring and control.

## 1. Introduction

The European Union produces about 67% of the world’s olive oil (about 2 million tons per year), and is the leading producer, consumer, and exporter of olive oil worldwide. The production of these 2 million tons of olive oil is distributed among three Mediterranean countries, namely Spain, Italy, and Greece, producing about 66, 15, and 13%, respectively, of the total EU production. In addition, olive oil is among the most common and important ingredients of the Mediterranean diet, Greece being the biggest consumer per capita among the EU countries, with about 12 kg per person per year [1]. According to the EU regulations [2], olive oils are categorized into eight different categories of olive oils and olive pomace oils: extra virgin olive oil, virgin olive oil, lampante virgin olive oil, refined olive oil, olive oil composed of refined olive oil and virgin olive oils, olive pomace oil, crude olive pomace oil, and refined olive pomace oil. Among these olive oils, only the extra virgin olive oil (EVOO), the virgin olive oil (VOO), the olive oil composed of refined olive oil and virgin olive oils, and the olive pomace oil can be purchased directly at the retail level, with the EVOOs and VOOs obtained directly and exclusively from olives and solely by mechanical means, resulting in a considerably increased production cost. In fact, these two categories of olive oil have the largest demand for edible purposes, and are considered as high-quality olive oils exhibiting a wide range of characteristics (e.g., taste, color, smell, feel), depending on the type of olive, the harvest date, the soil, the climatic conditions, and so forth. The increasing demand for EVOOs and VOOs, combined with authenticity requirements, oblige the related agricultural sector and industry to provide precise definitions of various parameters such as cultivar, geographical origin, agronomic and organoleptic methods, and production technology. In that view, the European Union established a quality policy (EC Regulations 2081/92 and 2082/92) [3,4] that incorporates the concepts of protected designations of origin (PDO) and protected geographical identification (PGI). A survey conducted by Errach et al. [5], concerning the importance of consumers’ choice of purchase of a PDO extra virgin olive oil, showed a significant preference to origin and, especially, the willingness to pay for a PDO-labelled olive oil. All these, together with the tendency of consumers to attribute higher importance to the quality rather than the quantity of foodstuffs, together with the potential health and therapeutic benefits associated with olive oil consumption, have led to increasing demand and to higher market values for EVOOs and VOOs. Regarding all the above, olive oil producers tend to adulterate virgin olive oil with less expensive edible oils, olive residue oils (refined or pomace olive oil), and other vegetable or seed oils such as corn, peanut, and sunflower [6,7]. In order to avoid such fraud actions, and for quality control reasons, different analytical methods have been proposed for the determination of traceability and authenticity of olive oil by studying the botanical or geographical origin. For these purposes, several parameters/compounds in the composition of olive oils are considered to be the study factors, and they are differentiated into major components (fatty acids and triglycerides) and minor components (sterols, phenolic compounds, pigments, hydrocarbons, and tocopherols), according to their presence in olive oils [8,9]. Ollivier et al. [10,11], to distinguish some French olive oils by their PDO, performed separate analyses of their fatty acid and triacylglycerol composition. The fatty acids content was analyzed by Gas Chromatography (GC), while triacylglycerols were analyzed by a High-Performance Liquid Chromatography (HPLC), considering a separation according to their specific carbon composition. Then, the discrimination of the olive oils was carried out with the help of a machine learning algorithm, Linear Discriminant Analysis (LDA). This study has demonstrated that these two techniques could successfully classify olive oils from the same country using their fatty acid and triacylglycerol compositions; however, only samples with different varietal compositions were perfectly differentiated, while olive oil samples from common varieties were misclassified. In another work by Bendini et al. [12], Fourier Transform Infrared spectroscopy (FTIR) was used for studying the free fatty acid content of 84 monovarietal olive oil samples originating from eight Italian regions. They succeeded to discriminate most of the samples using Principal Component Analysis (PCA). In the same way, Tapp et al. [13] and De Luca et al. [14] also applied FTIR spectroscopy for cluster analysis and the classification of 60 different olive oils originating from four European countries and Morocco, both reporting satisfactory results. Another analytical method used for the analysis of olive oils is Nuclear Magnetic Resonance (NMR) [15,16]. In the review of Dais et al. [17], useful information can be found covering various aspects of NMR analysis of olive oils, such as the NMR instrumentation, sample preparation, and statistical analysis of NMR data, as well as information regarding the geographical and botanical discrimination of olive oils. More recently, Gyftokostas et al. [18], employing Laser-Induced Breakdown Spectroscopy (LIBS) and different machine learning algorithms (e.g., Principal Component Analysis (PCA), Linear Discriminant Analysis (LDA), k-Nearest Neighbors (k-NN), and Support Vector Classifiers (SVC)) have successfully classified different Greek olive oils, based on their geographical origin, achieving classification accuracies up to 90%.

Laser-Induced Breakdown Spectroscopy (LIBS) is a laser-based technique of atomic emission spectroscopy (AES) that utilizes a laser-generated plasma as the vaporization, atomization, and excitation source. It should be noted that LIBS is regarded as an attractive technique and its popularity has considerably increased over the past few years, mainly due to its simplicity, the immediate delivery of qualitative and quantitative elemental analysis, and its ability to analyze all states of matter (i.e., solid, liquid, and gas) [19,20,21]. According to this technique, a focused laser beam induces a dielectric breakdown on a sample’s surface, leading to plasma formation. Due to the excited species present in the plasma (i.e., atoms, ions, etc.), emission of radiation occurs. The collection of the emitted radiation and its subsequent spectral analysis can provide valuable information about the elemental composition of the sample [22,23,24]. Because of the unique features exhibited by LIBS, that is, the absence of any time-consuming sample preparation or pre-treatment, the use of a very small amount of sample, the ability to perform in real time, in situ, and/or remotely, as well as its quasi-non-destructive character, the number of LIBS applications has dramatically increased over the last few years [25,26,27,28], including, more recently, its application for food analysis [29,30,31,32], and the analysis of olive oils as well [33,34,35].

On the other hand, absorption spectroscopy is probably the most common and simplest spectroscopic technique found in a lab for qualitative and quantitive identification, and/or the characterization of unknown compounds in a sample. Absorption spectroscopy is largely used in olive oil studies, mostly for the determination of an olive oil’s natural pigments (i.e., carotenoids, chlorophylls, and their derivatives), and their quantification, providing useful information about the quality and authenticity of the olive oil [36,37,38,39].

The present work is a continuation of systematic research conducted in our group, aiming (i) to investigate the applicability, the limitations, and the pros and cons of the application of LIBS (i.e., a laser-based emission spectroscopy technique), assisted by modern machine learning algorithms, for food security and quality control issues, and (ii) to evaluate and assess the suitability and efficiency of different machine learning algorithmic approaches for the successful classification of such spectroscopic data. In particular, in the present work, besides LIBS, the absorption spectra of olive oils in the visible region were also used and analyzed by the same machine learning algorithms as for the LIBS emission spectra, and for the classification of olive oil samples regarding their geographical origins. More specifically, the present work investigates the application of both Laser-Induced Breakdown Spectroscopy (LIBS) and absorption spectroscopy for the discrimination/classification of 170 olive oil samples (most of them characterized as extra virgin olive oils (EVOOs) and some of them as virgin olive oils (VOOs)) originating from three geographical regions of Greece, namely Crete, Lesvos, and Peloponnese, which are the major producers of olive oil in Greece. Among these samples, 27 binary mixtures of olive oils from these three regions were prepared and also studied in terms of their discrimination vis-à-vis the non-mixed olive oils, since a common problem of the olive oil market is the mislabeling of the geographical origin of EVOOs and VOOs, especially those bearing PDO and PGI marks, by mixing them with cheaper olive oils from different origins. To the best of our knowledge, it is the first time that emission and absorption spectra of the same samples have been analyzed by the same machine learning algorithms (i.e., Linear Discriminant Analysis (LDA) and Support Vector Classifier (SVC)). The determined accuracies obtained by the two experimental approaches and the different classification methodologies are presented, discussed, and compared.

## 2. Results

Olive oil is mainly composed of fatty acids, both saturated and unsaturated, in the form of triglycerides and several other constituents present in much smaller amounts (e.g., glycerol, phosphatides, pigments, flavor compounds, sterols, etc.). Among the unsaturated fatty acids, oleic (C_18_H_34_O_2_) and linoleic acids (C_18_H_32_O_2_) are the most abundant ones. In addition to the above, there are different pigments (being responsible for the olive oil color), such as carotenoids and chlorophyll, occurring in the form of carotene (C_40_H_56_), lutein (C_40_H_56_O_2_), chlorophyll A (C_55_H_72_O_5_N_4_Mg) and B (C_55_H_70_MgN_4_O_6_), and so forth. In general, olive oil’s composition varies with the geographical origin, climate, growing conditions, harvest time, and so forth.

**Figure 1:**
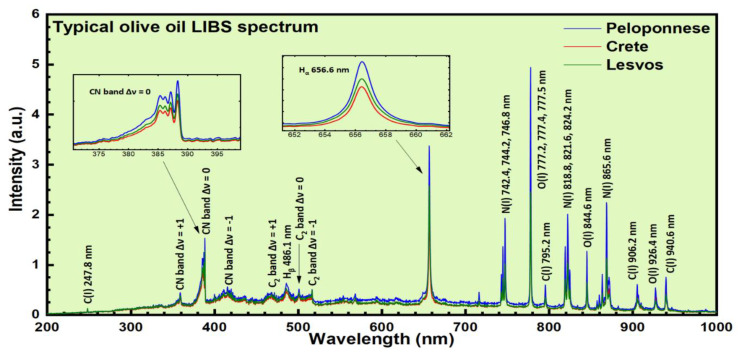
Some characteristic LIBS spectra of olive oils.

Due to the elemental composition of the major constituents of olive oil, the corresponding olive oil LIBS spectrum is expected to be dominated by the characteristic spectral features of elements such as hydrogen, oxygen, and carbon. As an example, in Figure 1, the atomic emissions of these elements are shown. As can be seen, a typical LIBS spectrum of an olive oil sample consists of atomic emissions of hydrogen’s Balmer series lines, Hβ at 486.1 nm and Hα at 656 nm, Oxygen O(I) spectral lines at about 777 nm, 844.6 nm, and 926.4 nm, Nitrogen N(I) spectral lines at 742 nm, 818 nm, and 865.6 nm, and Carbon C(I) spectral lines at 247.8 nm, 795.2 nm, 906.2 nm, and 940.6 nm. In addition, some molecular bands were also observed arising from molecular species formed under plasma conditions, for example, some bands corresponding to CN and C_2_ molecules. So, the molecular bands of the CN system were clearly observable at about 360 nm, 388 nm, and 422 nm, and attributed to the Δν = +1, Δν = 0 and Δν = −1 vibrational sequences. Also, the C_2_ Swan bands system appeared clearly at about 470 nm, 516 nm, and 559 nm, and attributed to the Δν = +1, Δν = 0 and Δν = −1 vibrational sequences. These molecules do not exist as independent molecular entities in olive oil, but they are either formed following some complicated chemistry paths under plasma conditions and/or can result from the fragmentation of larger organic molecules (e.g., fatty acids, etc.) present in the olive oil.

**Figure 2:**
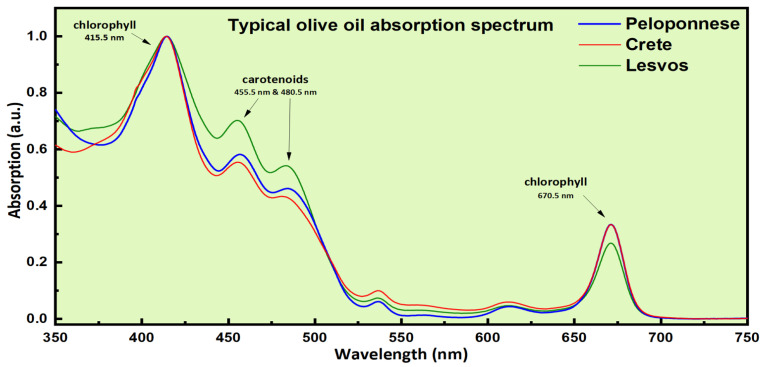
Some characteristic absorption spectra of olive oils.

In Figure 2, some representative absorption spectra of olive oil samples with different geographical origins are shown as an example. As shown, two chlorophyll-related absorption bands at 415.5 nm and 670.5 nm are prominently observed, while the two absorption bands at 455.5 nm and 480.5 nm are due to carotenoids.

From a simple inspection of the olive oils’ LIBS and absorption spectra presented in Figure 1 and Figure 2, respectively, it becomes apparent that they are very similar. This is more obvious in the case of the LIBS spectra, where their differences are mostly expressed in the variation of the relative intensities of the observed emissions. In the case of the absorption spectra, the major differences are mostly related to the carotenoid absorption bands, at 455.5 and 480.5 nm. For example, the olive oil samples from the island of Lesvos present larger absorption at these wavelengths compared to the olive oil samples originating from Peloponnese and the island of Crete.

### 2.1. LIBS Spectra Analysis by Linear Discriminant Analysis (LDA)

In the analysis presented in the next section, both LIBS and absorption spectra were initially preprocessed by means of the PCA algorithm for the reduction of the dimensions of the initial datasets since most of the initial spectral information carried by these emission and absorption spectra can be maintained in some Principal Components (PCs) only. Then, the preprocessed data were introduced into the LDA and SVC algorithms for the construction of the corresponding predictive models. It must be noted that the 27 binary mixtures prepared by mixing olive oils from Lesvos and Peloponnese (Les–Pel), Lesvos and Crete (Les–Cre), and Peloponnese and Crete (Pel–Cre) were considered as three classes (i.e., one for each pair of regions). Thus, along with the three classes of olive oils originating from Crete (Cre), Peloponnese (Pel), and Lesvos (Les), three more classes corresponding to the Les–Pel, Les–Cre and Pel–Cre mixtures, making six classes in total, were used for the algorithmic training (see also Section 4).

**Figure 3:**
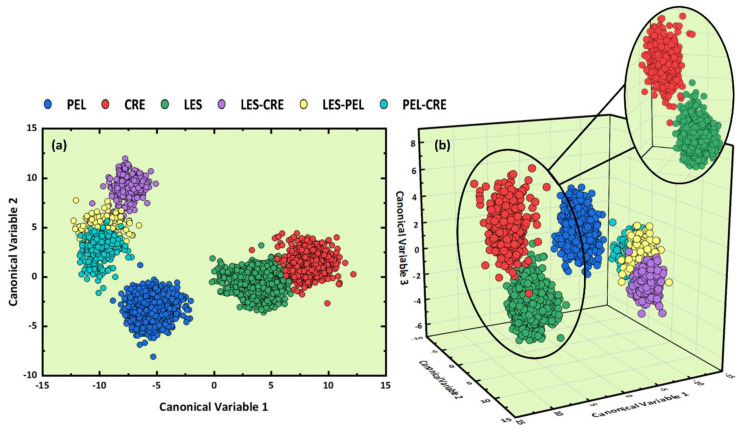
(**a**) 2D and (**b**) 3D LDA plots obtained using the olive oils’ LIBS spectra.

The results obtained from the analysis of the LIBS spectral data using the LDA algorithm are presented in Figure 3a,b. As can be seen in Figure 3a, the formation of six different clusters was observed, with some overlapping occurring between the Lesvos and Crete samples, and between the three clusters corresponding to the mixtures of the different regions. However, the slight overlapping of the Lesvos and Crete olive oil samples suggested by the 2D LDA representation is rather a weakness of the 2D representation, as evidenced by the 3D LDA plot in Figure 3b, indicating that these samples are rather well distinguished among them. Concerning the classification of the binary mixtures, it is important to notice that the 3D LDA plot reveals clearly that they are well separated as a whole from the classes of non-mixed samples (i.e., the Les, Cre, and Pel samples). The estimated accuracy of the predictive model was found to be (99.9 ± 0.1)%, via the “k-fold” cross-validation method, where k was set equal to 10 (k = 10).

At this point, it is a useful reminder that the 2D and/or 3D representations employed for visualization purposes are sometimes unsatisfactory for the presentation of the classification results due to the limited number of dimensions they use (i.e., two and three dimensions respectively). Thus, some contradictory situations can occur, for example, to have some degree of overlapping in the 2D and/or 3D visualizations, while the LDA analysis provides high estimated accuracies. Such situations can be better understood if one considers that for the training of the LDA model the maximum number of canonical variables was used (i.e., five canonical variables in the present case). This situation can result in some truncation of the information contained in the 2D and 3D plots, without, however, affecting the results of the predictive model.

**Figure 4:**
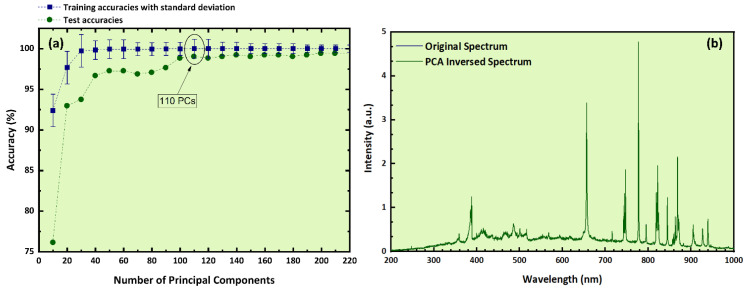
(**a**) Training and test accuracies as a function of the number of PCs, (**b**) experimental LIBS spectrum and PCA-inversed spectrum.

Despite the high accuracy of the LDA predictive model calculated via the cross-validation method, to further ensure the robustness and effectiveness of the model, an external validation procedure was applied as well. In that view, the initial dataset was divided into training and test sets, the former consisting of 153 samples while the latter comprising the remaining 17 samples (i.e., 4 samples from Crete, 4 samples from Lesvos, 6 samples from Peloponnese, and one sample from each binary mixture category). So, the PCA algorithm was first applied, leading to an optimum number of 110 PCs. Figure 4a illustrates the training accuracies, along with standard deviation and test accuracies, as a function of the number of PCs employed (for more details, see also Section 4). As it is shown, for the first 90 PCs, the test accuracy was significantly lower than the training accuracy, suggesting that the information contained in these 90 PCs was not enough for the construction of a predictive model. Regarding this issue, 100 PCs seem to be a better choice, as training and test accuracies were determined to be very close. However, test accuracy was not within the standard deviation of the model, a situation indicating some overfitting of the model. For that reason, 110 PCs (explaining more than 99% of the original data variance) were chosen, as in this case, both training and test accuracies were found to be very close and very high, attaining the values of (100 ± 0.9)% and 99.1% respectively. These results indicate a very well-trained algorithm. For further confirmation of these findings, the inversed spectrum was reconstructed (see also Section 4), using the 110 PCs employed for the training as well, and is displayed together with the original experimental LIBS spectrum. As can be seen, the two spectra are practically identical, suggesting that there is no observable lack of the initial information.

For further evaluation of the trained model, the classification report and confusion matrix were constructed and are presented in Table 1a and Table 2b, respectively (see also Section 4). The obtained “f1-score” along with the estimated “accuracy” and “weighted average” had values of 1 (or 100%), thus confirming the above-mentioned successful results. From the corresponding confusion matrix (see Table 1b), all spectra were found to be correctly classified, except for two samples of the Les–Pel class that were identified as belonging to the Pel–Cre class, and one sample of the Pel class, which was misclassified as a Les sample. Furthermore, it was quite important that none of the binary mixtures were misclassified, all having been correctly separated from the Cre, Les, and Pel samples. These findings clearly suggest that the LDA algorithm can be successfully used for the discrimination of olive oil samples regarding their geographical origin.

### 2.2. LIBS Data Analysis by the Support Vector Classifier (SVC)

**Figure 5:**
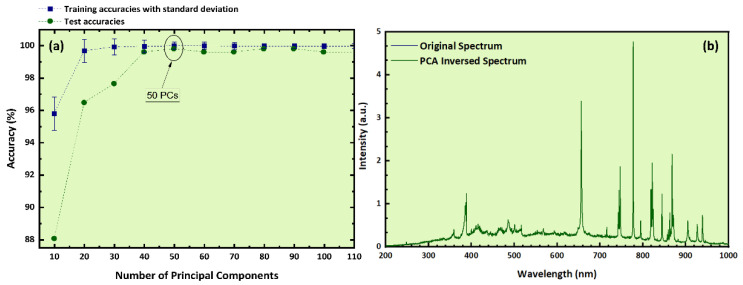
(**a**) Training and test accuracies as a function of the number of PCs, (**b**) experimental LIBS spectrum and PCA-inversed spectrum.

Next, the LIBS spectroscopic data were analyzed by the SVC algorithm after having been preprocessed by means of PCA. In this case, the optimum number of PCs was estimated to be 50 (Figure 5a), explaining more than 99% of the original data variance. The determined training accuracy with cross-validation was found to attain (100 ± 0.8)%. Again, to ensure the effectiveness and prediction capability of the algorithm, an external validation procedure was performed. The obtained training accuracy reached as high as (100 ± 0.4)%. The classification report of this model is presented in Table 2a. The “f1”, “accuracy”, and “weighted average” scores ascended to a value of 1, denoting the successful training of the algorithm. Most importantly, the test accuracy was up to 99.8%, which is quite high and inside the standard deviation of the algorithm, demonstrating the efficiency of the predictive model. This is also reflected in the confusion matrix (see Table 2b), which displays that only two samples were misclassified, whereas all the others were categorized correctly. In more detail, only one Les and one Les–Pel samples were falsely predicted as Cre and Pel–Cre samples, respectively. As far as it concerns the binary mixtures, they were all predicted correctly. In Figure 5b, the reconstructed spectrum calculated using 50 PCs is presented, along with the experimental LIBS spectrum. As can be seen, no observable differences between these spectra are apparent, indicating that most of the initial information carried by the entire spectrum (consisting of 2754 data points, i.e., wavelengths) was maintained by using only 50 PCs. The original dataset was reduced approximately 55 times without affecting the performance of the predictive model. The most significant features derived from the discrimination procedure via SVC and the PCA loadings for the first three PCs are presented in the Supplementary Material, in Figures S1 and S2, respectively (see also Section 4.3 for further information).

### 2.3. Absorption Data Analysis by Linear Discriminant Analysis (LDA)

The same methodology applied to the LIBS spectra was also followed for the analysis of the absorption spectra. Thus, after prepossessing the original data via PCA, they were introduced to the LDA and SVC algorithms for the construction of the corresponding predictive models.

**Figure 6:**
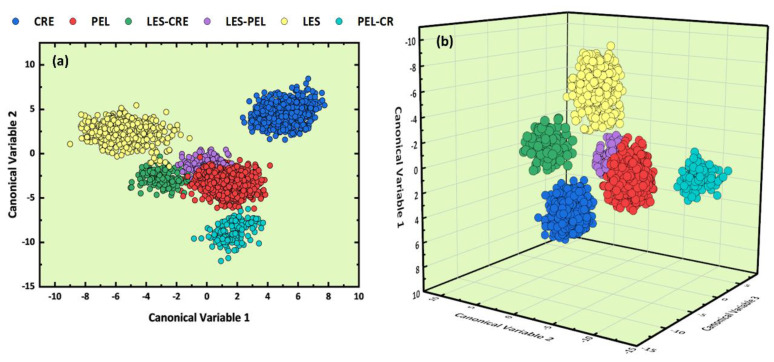
2D (**a**) and 3D (**b**) LDA plots obtained using the olive oils’ absorption spectra.

The resulting 2D and 3D LDA graphs are displayed in Figure 6a,b, respectively. From inspection of Figure 6a, only three clusters seem to be clearly distinguishable, corresponding to the Cre, Les, and Pel–Cre samples, while the formation of a fourth cluster containing the Pel, Les–Pel, and Les–Cre samples is noticeable as well. However, this overlapping disappears in the 3D LDA graph of Figure 6b, where only some overlap between the Pel and Les–Pel samples is observable. The estimated cross-validation accuracy reached a value of (100 ± 0.0)%. Again, the inconsistency between the high estimated accuracy and the overlapping shown in the 2D and 3D LDA plots, as mentioned previously, is due to the limited capabilities of visualization by such plots and the five PCs used for training purposes.

**Figure 7:**
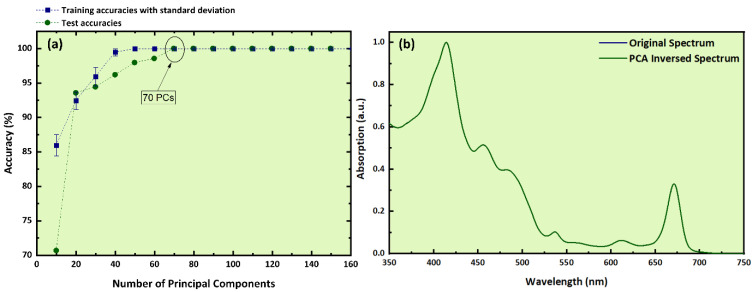
(**a**) Training and test accuracies as a function of the number of PCs, (**b**) experimental absorption spectrum and PCA-inversed spectrum.

Next, the optimum number of PCs was determined; it was found that 70 PCs can explain more than 99% of the original variance (see Figure 7a), while the corresponding trained model achieved a quite high training accuracy (with low standard deviation) and testing accuracy (within the margins of standard deviation). For the implementation of the external validation, the initial dataset was divided into 153 samples for training and 17 samples for testing, similarly to what had been done for the LIBS dataset. Next, the PCA-preprocessed data were imported into the LDA algorithm, which resulted in a training accuracy of (100 ± 0.0)% for the external validation procedure.

The “f1-score”, the “accuracy”, and the “weighted average” were found attaining values of 1 (or 100%), while the test accuracy reached a value of 100%, suggesting again an exceptionally well-trained algorithm. These findings are summarized in Table 3a. This result was further verified by constructing the confusion matrix (see Table 3b), with all samples being correctly predicted, ensuring again the effectiveness and robustness of the LDA-constructed predictive model. Finally, the reconstructed spectrum was calculated using the 70 PCs and is presented in Figure 7b, together with the original absorption spectrum. As can be seen, the two spectra are practically identical, despite the reduction of the original dataset by approximately 11 times (i.e., using 70 PCs instead of 801 wavelengths).

### 2.4. Absorption Data Analysis by the Support Vector Classifier (SVC)

**Figure 8:**
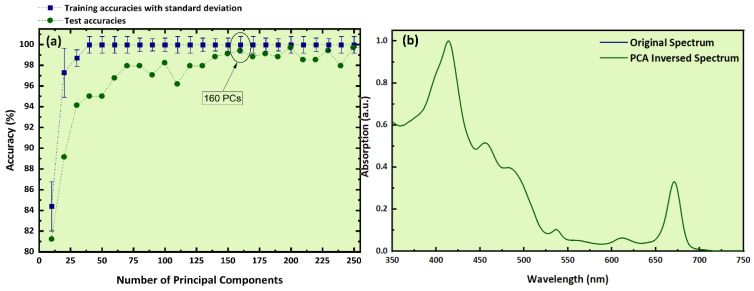
(**a**) Training and test accuracies as a function of the number of PCs, (**b**) experimental absorption spectrum and PCA-inversed spectrum.

Finally, the SVC algorithm was applied to the absorption spectra after preprocessing them by PCA. In this case, the optimum number of PCs was determined to be 160 (see Figure 8a) with explained variance exceeding 99%. The determined training accuracy using the cross-validation method was found to be (99.1 ± 0.8)%. Then, for the evaluation of the constructed model, an external validation procedure was performed. For that, a training and a test set were created, similarly to the previous cases. The constructed predictive model resulted in an estimated training accuracy of (99.9 ± 0.6)% and a test accuracy of 99.4%. The corresponding classification report is presented in Table 4a. As shown, the “f1-score”, the “accuracy”, and “weighted average” values were found attaining a value of 1 (or 100%). For further evaluation, the corresponding confusion matrix is shown in Table 4b. As can be seen, only six Pel samples were misclassified, two of them misidentified as Cre, and four as Les samples. It is worth adding here that all mixtures were correctly classified.

Furthermore, the calculated reconstructed spectra were found to be identical to the experimental absorption ones, as can be seen in Figure 8b. It must be emphasized that for all predictive models constructed (i.e., using the LIBS and the absorption spectra), the same number of PCs were found to be the optimum for both internal (cross-validation) and external validation, but only the results for external validation are presented here since they are the most important, considering that they validate the real performance and effectiveness of the predictive models. The most significant features derived from the discrimination procedure by SVC and the PCA loadings for the first three PCs are presented in the Supplementary Materials in Figures S3 and S4, respectively (see also Section 4.3 for further information).

## 3. Discussion

The obtained accuracies from all the predictive models and for both LIBS and absorption spectroscopic data are summarized in Table 5. As can be seen, the training accuracies for all the constructed models exceeded 99% by the reduction of the original data, from approximately 5 times, in the case of SVC algorithm with absorption data, to the extraordinary value of 55 times, in the case of SVC model for the LIBS data. One of the most important results of the present work is the test accuracies attained via external validation. These accuracies reached percentages as high as 100%, verifying the excellent effectiveness and robustness of the constructed models for all studied cases. Thus, the discrimination of olive oils (EVVOs and VOOs) based on their geographical origin, and the discrimination of mixtures of these olive oils from the unmixed ones was accomplished with great success using both LIBS and absorption spectroscopy.

In more detail, the optimum numbers of PCs for the LIBS data were determined to be 110 and 50, for the LDA and SVC algorithms, respectively. The corresponding figures for the absorption data were determined to be 70 and 160, for the LDA and SVC algorithms, respectively. These findings are quite interesting, especially considering that the LIBS spectra had 2754 dimensions, and the absorption spectra had 801 dimensions, while it was expected that fewer PCs would be enough for the construction of predictive models in the case of the absorption data (since, for the proper choice of PCs, the dimension of the initial dataset is critical). Thus, since PCA is an algorithm that can be applied effectively mostly in linear data, it seems that the LIBS spectroscopic data exhibited higher linear correlations between them than the absorption data and, as a result, a specific number of PCs could contain a higher amount of the initial information in the case of LIBS spectra than in the case of absorption spectra. Furthermore, the high accuracies obtained using the absorption data indicate that these data should also present some degree of linear correlations, but probably not as strong as the linear correlation degree existing in the LIBS data. This is a qualitative finding and is somewhat puzzling, as the absorption spectra are generally thought of as possessing more “linear correlation” character than the emission spectra of the laser-produced plasma, the latter being a rather stochastic process. This issue, concerning the nature of the spectroscopic information (i.e., emission and absorption spectra) and its relation to the machine learning algorithms and the corresponding predictive models, seems quite interesting and needs further exploration. However, it is beyond the scope of the present work. Nevertheless, both spectroscopic techniques indicate that linear algorithms seem to operate more efficiently for discrimination/classification purposes, and that the more suitable algorithmic models for discrimination are probably the linear ones.

In addition, from the comparison of the original and PCA-reconstructed spectra, it is obvious that most of the initial data information was maintained, while the initial data size was significantly reduced. Also, the explained variance for all the predictive models exceeded 99%, verifying the similarity of the original and inversed spectra. Furthermore, the present work has shown that these high percentages of explained variance were obligatory for the construction of robust and effective predictive models, indicating that the spectra have a quite high signal to noise ratio (SNR) for both LIBS and absorption spectroscopic spectra (see Section 4 for further information about the PCA algorithm). This is very important, especially in the case of LIBS, verifying the proper conduction of the experimental procedure, in which the correct alignment, along with the chosen temporal conditions, are of great importance for the collection of a signal with high SNR from the emitted plasma radiation.

To facilitate the presentation of the obtained classification by means of visualization, the corresponding 2D and 3D LDA plots were constructed for both types of spectroscopic data. For the further evaluation of the predictive models, the k-fold cross-validation method was applied, with success rates larger than 99% for both LIBS and absorption spectra. Moreover, external validation was performed and used as an additional and necessary validation tool for the proof of the effectiveness and robustness of the predictive models. The most important result was that the test accuracies, which determine the prediction performance of the algorithms, reached values as high as 100% for both spectroscopic techniques, indicating the outstanding performance of the predictive models for discrimination purposes. In addition, all the constructed predictive models were found succeeding to perfectly discriminate the mixtures (i.e., Les–Cre, Les–Pel, and Pel–Cre) from the unmixed olive oils from the different regions (i.e., Les, Cre, and Pel), this being among the main goals of the present work. Thus, these findings imply not only the successful discrimination of EVOOs and VOOs from mixed samples within this work, but also the adequacy of LIBS and absorption spectroscopy in such a task. This issue requires further investigation and is a matter for future work.

In conclusion, the present work indicates that both the emission spectra of LIBS and the absorption spectra, both assisted by LDA or SVC machine learning algorithms, can operate very efficiently, providing significant information for the discrimination/classification in terms of the geographical origin of olive oil.

## 4. Materials and Methods

### 4.1. Olive Oil Samples

In total, 170 olive oil samples were studied, comprising 143 olive oil samples and 27 mixtures of them. They were collected from three different regions of Greece, that is, the islands of Lesvos and Crete, and from different places of Peloponnese. More specifically, 49 samples originated from Lesvos (Les), 40 from Crete (Cre), and 54 from Peloponnese (Pel). The 27 binary mixtures were prepared by mixing olive oils from these three geographical regions to investigate if they could be discriminated from the non-mixed ones. To that purpose, mixtures with various mixing ratios ranging from 10% to 90% were used, namely 9 mixtures of olive oils from Lesvos and Crete (Les–Cre), 9 mixtures of olive oils from Lesvos and Peloponnese (Les–Pel) and 9 mixtures of olive oils from Peloponnese and Crete (Pel–Cre). All the olive oils were collected from local producers within the frame of the Emblematic Action “The Olive Road” (see also the Acknowledgements). For their preservation, all samples were stored in a refrigerator in dark glass bottles, at a temperature of −2 to −4 °C to avoid oxidation. Prior to the experiments, the samples were left at room temperature for 24 h. For the LIBS measurements, 2 mL of the olive oil samples were placed in small shallow glass recipients, while, for the absorption measurements, the olive oil samples were placed in 1 mm high-quality glass cells. Some representative photos of the samples are shown in Figure S5 in the Supplementary Material.

### 4.2. Experimental Setups

**Figure 9:**
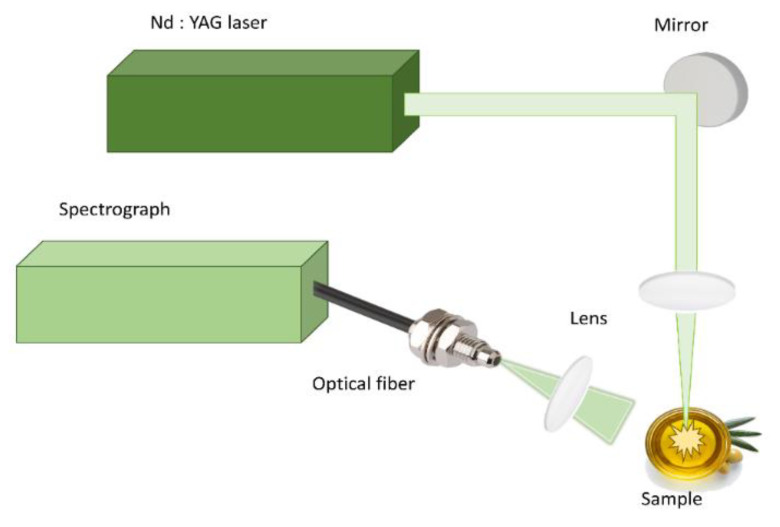
Schematic of the LIBS experimental setup.

For the LIBS experiments, a 5 ns Q-switched Nd: YAG laser (Quanta-Ray INDI) (Spectra-Physics Inc., Mountain View, CA, USA), operating at each fundamental wavelength at 1064 nm at a repetition rate of 1–5 Hz, was used. The laser energy was set at about 90 mJ per pulse to ensure a good signal-to-noise ratio (SNR) and to keep splashing from the liquid sample surface to a minimum. The laser beam was focused by a 150 mm focal length lens perpendicular to the sample’s free surface to induce the plasma. The emitted light from the plasma was collected by means of a 50 mm quartz lens and was introduced to an optical fiber bundle attached to the entrance slit of a portable spectrograph (AvaSpec-ULS4096CL-EVO, 75 mm focal length) (Avantes BV, Apeldoorn, The Netherlands) for spectral analysis. The spectrometer was equipped with an entrance slit of 10 μm, a 300 lines/mm diffraction grating, and a 4096 pixels CMOS detector, covering the spectral region from 185 to 1347 nm. For the need of the present investigation, the acquired spectra were recorded from 200 to 1000 nm, corresponding to 2754 pixels of the detector. A time delay (t_d_) of 1.28 μs, and a gate width (t_w_) of 1.05 ms were used for the temporal gating of the detector. For better statistics, 10 consecutive laser shots were averaged, corresponding with one LIBS measurement, while 30 such LIBS measurements were taken for each sample at different positions on its surface. A schematic representation of the LIBS experimental setup is shown in Figure 9, while further information regarding the experimental conditions can be found in [33].

The absorption measurements were performed using a UV-VIS-NIR spectrophotometer (Jasco V-670) (Jasco Deutschland GmbH, Pfungstadt, Germany). The spectral range of interest for the present olive oil measurements was set from 350 to 750 nm. Each olive oil absorption spectrum used in this study was the average of 20 consecutive absorption spectra. Each absorption spectrum consisted of 801 data points (i.e., wavelengths).

### 4.3. Data Analysis

For the analysis of the obtained spectroscopic data, some of the most popular and widely used machine learning techniques were applied, namely Principal Component Analysis (PCA) [40], Linear Discriminant Analysis (LDA) [41], and Support Vector Classifier (SVC) [42]. PCA is an unsupervised technique, not requiring any a priori knowledge of the data labels, and is usually employed for pattern recognition (similarities and/or differences) among multivariate/multidimensional data. PCA is also used for dimensionality reduction purposes, as it is capable of retaining most of the initial information of the dataset. The LIBS and the absorption spectra are typical examples of such multidimensional data, having many dimensions corresponding to the pixels of the light detector used for the recording of the spectrally analyzed light provided by some spectrograph. So, PCA can achieve the efficient transforming of the original correlated data into a new set of uncorrelated variables, namely Principal Components (PCs), based on the data variance (e.g., the first Principal Component (PC1) is a linear combination of all actual variables in such a way that it has the greatest range of variation; PC2 has the next major amount of variation, and so on). These new variables can be displayed graphically, allowing the original data set to be visualized in two and/or three dimensions. Moreover, the PCA algorithm can perform as a noise removal technique, since it can maintain only the features (i.e., wavelengths) carrying the larger variation of the original data, and the principal components with low variance (e.g., representing the noise of the spectra) can be discarded. In addition, the loadings derived from PCA analysis are presented. The loadings indicate the features (e.g., wavelengths) with the greater variance for each PC. In both the LIBS and absorption spectroscopic data, the maximum variance corresponds to the peaks (e.g., emission and absorption peaks), which contain most of the information.

Contrary to the PCA algorithm, LDA is a supervised technique and requires prior knowledge of data labels, which means that the corresponding label for each sample category is used as an input for this algorithm. LDA aims to achieve maximum differentiation between classes and, at the same time, minimum deviation between the data within each class, through a classifier with a linear decision boundary. It can be used, also, for dimensional reduction and visualization purposes of multidimensional data.

The Support Vector Machine (SVM) algorithm is also a supervised technique and can be applied both for regression and classification tasks. The objective of the Support Vector Classifier algorithm is to find a hyperplane in an N-dimensional space that uniquely classifies the data points. Then, it projects the data points onto the hyperplane in such a way that the clusters separate with the greatest amount of margin. This algorithm also has the ability to compute coefficients (e.g., statistical weights) that correspond to the most significant features (e.g., wavelengths), which contribute the most during the discrimination procedure. SVC is suitable both for linear and nonlinear data. In the present work, both LDA and SVC were used for the construction of predictive models.

The constructed LDA and SVC prediction models, for both the LIBS and absorption spectra, were then evaluated using the “k-fold” cross-validation method (in this work, k = 10). According to this method, the original data set is divided into k equal parts. Of the k-parts, one of them is used as validation data and for model testing, while the remaining k − 1 sections are used for the algorithmic training. The cross-validation process is then repeated k times and the results can be averaged to obtain a single estimation, that is, the predictive accuracy of the model along with the standard deviation. For a better evaluation of the predictive models, some more metrics were also computed, such as confusion matrices and classification reports. The former is a specific table layout, used to verify how much of the experimental data (spectra) were classified correctly. In this table, the rows represent the actual classes of the samples and the columns represent the classes predicted by the model. Its diagonal elements indicate the number of spectra that were predicted successfully. The latter metrics contain information about all the samples during the algorithmic training and provide a more detailed picture of the training process. Particularly, they give information about the “precision”, which represents a fraction of true positive values to the sum of true and false positive values, the “recall”, which represents a fraction of true positive values to the sum of true positive and false negative values, and the “f1-score”, which is the harmonic mean of the “precision” and “recall”, and its value range is between 0 and 1 (values above 0.5 can be considered successful). In addition, the “weighted average” was calculated, which takes into account the varying number of the samples that are contained in the different classes, and each number in the dataset is multiplied by a weight corresponding to the number of samples contained in each class before the final calculation is made. This metric is more precise from the “simple average” when the classes are imbalanced (e.g., 9 samples for each mixture class, and 40 samples for Crete, 49 for Lesvos, and 54 for Peloponnese). Lastly, the “support”, which indicates the number of actual occurrences of the class in the dataset, was computed.

Finally, and very importantly, external validation was performed for all the constructed predictive models applied for both LIBS and absorption data to ensure their robustness and validity. The importance of such external validation is based on the fact that a certain number of samples were totally removed from the training procedure and were used only for prediction. It must be emphasized that external validation is a necessary prerequisite procedure for the unambiguous evaluation of the real performance of a predictive model. Unfortunately, in several research works, it is often missing, thus reducing the credibility of the reported results. During this procedure, different numbers of PCs were used, and the different training and test scores were computed for each one and the results were visualized. Then, the minimum number of PCs, which resulted in a high training accuracy with low standard deviation, and a testing accuracy within the standard deviation, were selected as the optimum for the predictive model. Additionally, for the number of PCs that were selected as optimum, the PCA-reconstructed spectra were computed in order to inspect visually if any differences (i.e., possible loss of spectral information) occurred between the reconstructed and the original spectra. For all the analysis procedures, Python programming language, along with the scikit-learn machine learn package, were used [43].

For the utilization of the machine learning algorithms, spectra from 153 olive oil samples were used for training the algorithms and 17 for external validation. In that sense, the LIBS raw dataset for training consisted of a data matrix with dimensions of 4590 rows and 2754 columns, while the external validation dataset consisted of a data matrix with dimensions of 510 rows and 2754 columns. The absorption raw dataset for training consisted of a data matrix with dimensions of 3060 rows and 801 columns, while the external validation dataset consisted of a data matrix with dimensions of 340 rows and 801 columns.

## 5. Conclusions

In the present work, two different spectroscopic techniques, LIBS and absorption spectroscopy, were applied, and their efficiency for the classification/discrimination of Greek EVOOs and VOOs, in terms of their geographical origin, were evaluated. The former technique (i.e., LIBS) provided the visible emission spectrum (in the 200–1000 nm spectral region) of an olive oil sample, while the latter one (i.e., absorption spectroscopy) provided the absorption spectrum (350–750 nm). The emission lines of the LIBS spectra revealed the elemental composition of the olive oil sample, while the absorption bands within the 350–750 nm spectral region provided information regarding chlorophylls and carotenoids. It is a useful reminder that nowadays, the instrumentation of both techniques has reached a high level of maturity, with decreasing operational costs, and with the absorption spectroscopy being much more widely spread and routinely used in labs. Both spectroscopic techniques were applied independently on the same olive oil samples, and the resulting spectra were used for the construction of predictive models. In total, 170 olive oil samples were studied, with 143 samples originating from Lesvos, Crete, and Peloponnese, and the remaining 27 samples were binary mixtures of them, with different mixing ratios, prepared separately. Both LIBS and absorption spectroscopic data were at first preprocessed using the PCA algorithm to reduce the original dataset dimensions and, most importantly, to select a small number of PCs that contained the maximum initial information necessary for the construction of robust predictive models. The original datasets were reduced by more than 10 times. The produced reduced dimensions datasets were subsequently processed by the LDA and SVC algorithms, which showed that all studied samples could be successfully discriminated in four clusters, three corresponding to their geographical origin, and the fourth denoting the mixtures. Similar behavior regarding the formation of clusters of olive oil samples from different geographical regions has been also reported in some other works using different analytical techniques (e.g., FTIR, NMR, etc.), and multivariate analysis and statistical models for classification [11,13,14,15]). It is important to add that the dimensionality reduction of the original spectroscopic data did not affect the discrimination performance of the constructed predictive models. This can be very important since smaller datasets maintaining most of the initial information of the much larger initial datasets can be used for faster training, while allowing for the implementation of less sophisticated and expensive hardware. In general, both LIBS and absorption spectroscopy are relatively simple in terms of their instrumentation, while they allow for rapid and portable spectra acquisition, making them attractive tools for food authentication. Their implementation for geographic discrimination of olive oils resulted in excellent results in the present study, and strongly encourages further research and assessment of other machine learning algorithmic approaches as well.

## Figures and Tables

**Table 1:** (**a**) Classification report and (**b**) confusion matrix for LDA external validation.

(a) Classification Report			(b) Confusion Matrix							
	**f1-Score**	**Support**			**Predicted Values**					
**CRE**	1	87			**CRE**	**LES**	**LES–CRE**	**LES–PEL**	**PEL**	**PEL–CRE**
**LES**	1	143	**Actual Values**	**CRE**	120	0	0	0	0	0
**LES–CRE**	1	36		**LES**	0	120	0	0	0	0
**LES–PEL**	1	20		**LES–CRE**	0	0	30	0	0	0
**PEL**	1	145		**LES–PEL**	0	0	0	28	0	2
**PEL–CRE**	1	28		**PEL**	0	1	0	0	179	0
**Accuracy**	1			**PEL–CRE**	0	0	0	0	0	30
**Weighted avg**	1	459								

**Table 2:** (**a**) Classification report and (**b**) confusion matrix for SVC external validation.

(a) Classification Report			(b) Confusion Matrix							
	**f1-Score**	**Support**			**Predicted Values**					
**CRE**	1	203			**CRE**	**LES**	**LES–CRE**	**LES–PEL**	**PEL**	**PEL–CRE**
**LES**	1	275	**Actual Values**	**CRE**	120	0	0	0	0	0
**LES–CRE**	1	63		**LES**	1	119	0	0	0	0
**LES–PEL**	1	42		**LES–CRE**	0	0	30	0	0	0
**PEL**	1	277		**LES–PEL**	0	0	0	29	0	1
**PEL–CRE**	1	58		**PEL**	0	0	0	0	180	0
**Accuracy**	1			**PEL–CRE**	0	0	0	0	0	30
**Weighted avg**	1	918								

**Table 3:** (**a**) Classification report and (**b**) confusion matrix for the LDA external validation.

(a) Classification Report			(b) Confusion Matrix							
	**f1-Score**	**Support**			**Predicted Values**					
**CRE**	1	63			**CRE**	**LES**	**LES–CRE**	**LES–PEL**	**PEL**	**PEL–CRE**
**LES**	1	97	**Actual Values**	**CRE**	80	0	0	0	0	0
**LES–CRE**	1	14		**LES**	0	80	0	0	0	0
**LES–PEL**	1	20		**LES–CRE**	0	0	20	0	0	0
**PEL**	1	98		**LES–PEL**	0	0	0	20	0	0
**PEL–CRE**	1	14		**PEL**	0	0	0	0	120	0
**Accuracy**	1			**PEL–CRE**	0	0	0	0	0	20
**Weighted avg**	1	306								

**Table 4:** (**a**) Classification report and (**b**) confusion matrix for SVC external validation.

(a) Classification Report			(b) Confusion Matrix							
	**f1-Score**	**Support**			**Predicted Values**					
**CRE**	1	150			**CRE**	**LES**	**LES–CRE**	**LES–PEL**	**PEL**	**PEL–CRE**
**LES**	1	185	**Actual Values**	**CRE**	80	0	0	0	0	0
**LES–CRE**	1	25		**LES**	0	80	0	0	0	0
**LES–PEL**	1	34		**LES–CRE**	0	0	20	0	0	0
**PEL**	1	189		**LES–PEL**	0	0	0	20	0	0
**PEL–CRE**	1	29		**PEL**	2	4	0	0	112	0
**Accuracy**	1	1		**PEL–CRE**	0	0	0	0	0	20
**Weighted avg**	1	612								

**Table 5:** Summary of the algorithmic accuracies.

Spectroscopic Techniques:	LIBS				Absorption			
**Algorithmic Models:**	**LDA**		**SVC**		**LDA**		**SVC**	
**Number of PCs:**	110		50		70		160	
**Accuracies (%):**	**Training**	**Test**	**Training**	**Test**	**Training**	**Test**	**Training**	**Test**
	100 ± 0.9	99.1	100 ± 0.4.	99.8	100	100	99.9 ± 0.6	99.4

## Data Availability

Not applicable.

## Supplementary Materials

The following are available online, Figure S1: SVC coefficients for the LIBS predictive model, Figure S2: Loadings derived from PCA for LIBS data, Figure S3: SVC coefficients for the absorption predictive model, Figure S4: Loadings derived from PCA for absorption data, Figure S5: Samples from (a) Peloponnese, (b) Crete, (c) Lesvos, and (d) Lesvos–Crete mixture.

## Sample Availability

Samples are not available from the authors.
