# Supplementary material for: Classification of Greek Olive Oils from Different Regions by Machine Learning-Aided Laser-Induced Breakdown Spectroscopy and Absorption Spectroscopy
Source: Molecules. 2021 Feb 25;26(5):1241. doi: 10.3390/molecules26051241 (PMC7956679; doi:10.3390/molecules26051241)
Supplement: Supplementary file 1 [file molecules-26-01241-s001.zip › molecules-1085903-supplementary/molecules-1085903-supplementary final.pdf]

molecules

MDPI

Article

Classification of Greek olive oils from different regions by machine learning-aided Laser-Induced Breakdown Spectroscopy and Absorption Spectroscopy

Nikolaos Gyftokostas[1,2], Eleni Nanou[1,2], Dimitrios Stefas[1,2], Vasileios Kokkinos[3], Christos Bouras[3] and Stelios Couris[1,2,*]

[1] Department of Physics, University of Patras, 26504 Patras, Greece
[2] Institute of Chemical Engineering Sciences (ICE-HT), Foundation for Research and Technology-Hellas (FORTH), Patras, Greece
[3] Department of Computer Engineering & Informatics, University of Patras, 26504 Patras, Greece
* Correspondence: couris@upatras.gr

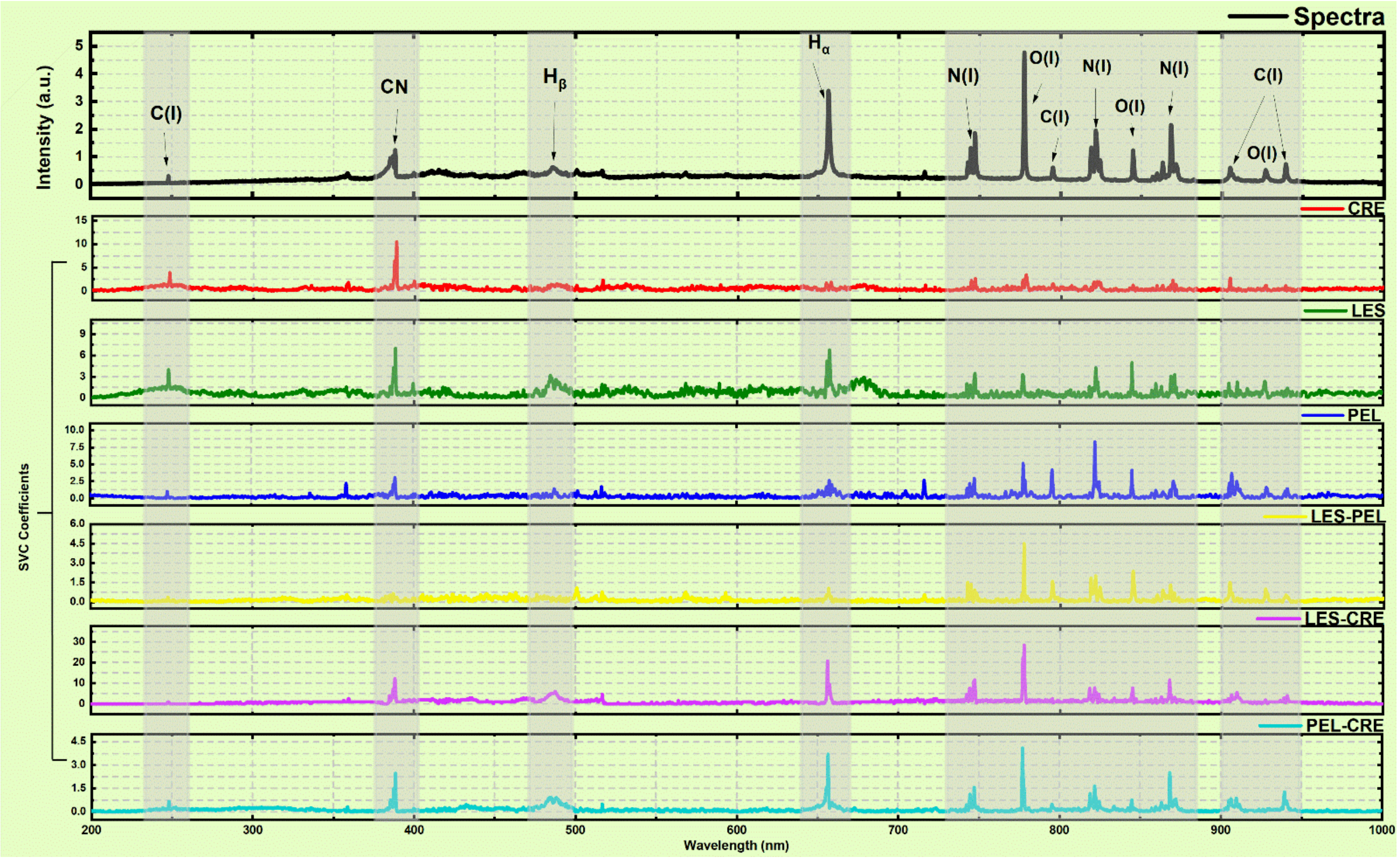

Figure S1. SVC coefficients for the LIBS predictive model, in comparison with a LIBS spectrum (black line). Coefficients correspond to the samples from Crete (CRE, red line), Lesvos (LES, green line) and Peloponnese (PEL, blue line), as well as the mixtures of LES-PEL (yellow line), LES-CRE (purple line) and PEL-CRE (light blue line).

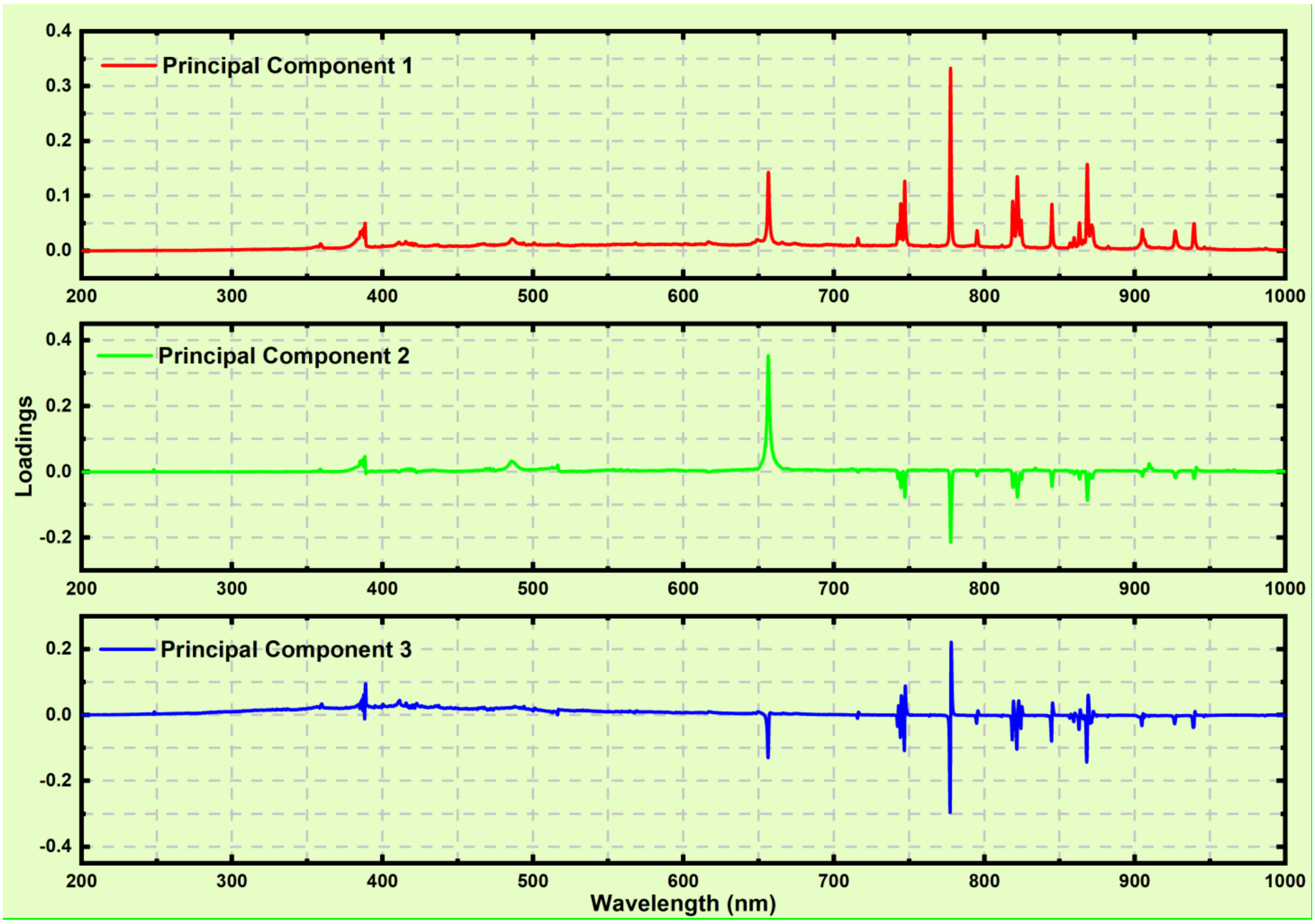

Figure S2. Loadings derived from PCA for LIBS data. The red line corresponds to the first Principal Component (PC), the green corresponds to the second PC and the blue line corresponds to the third PC.

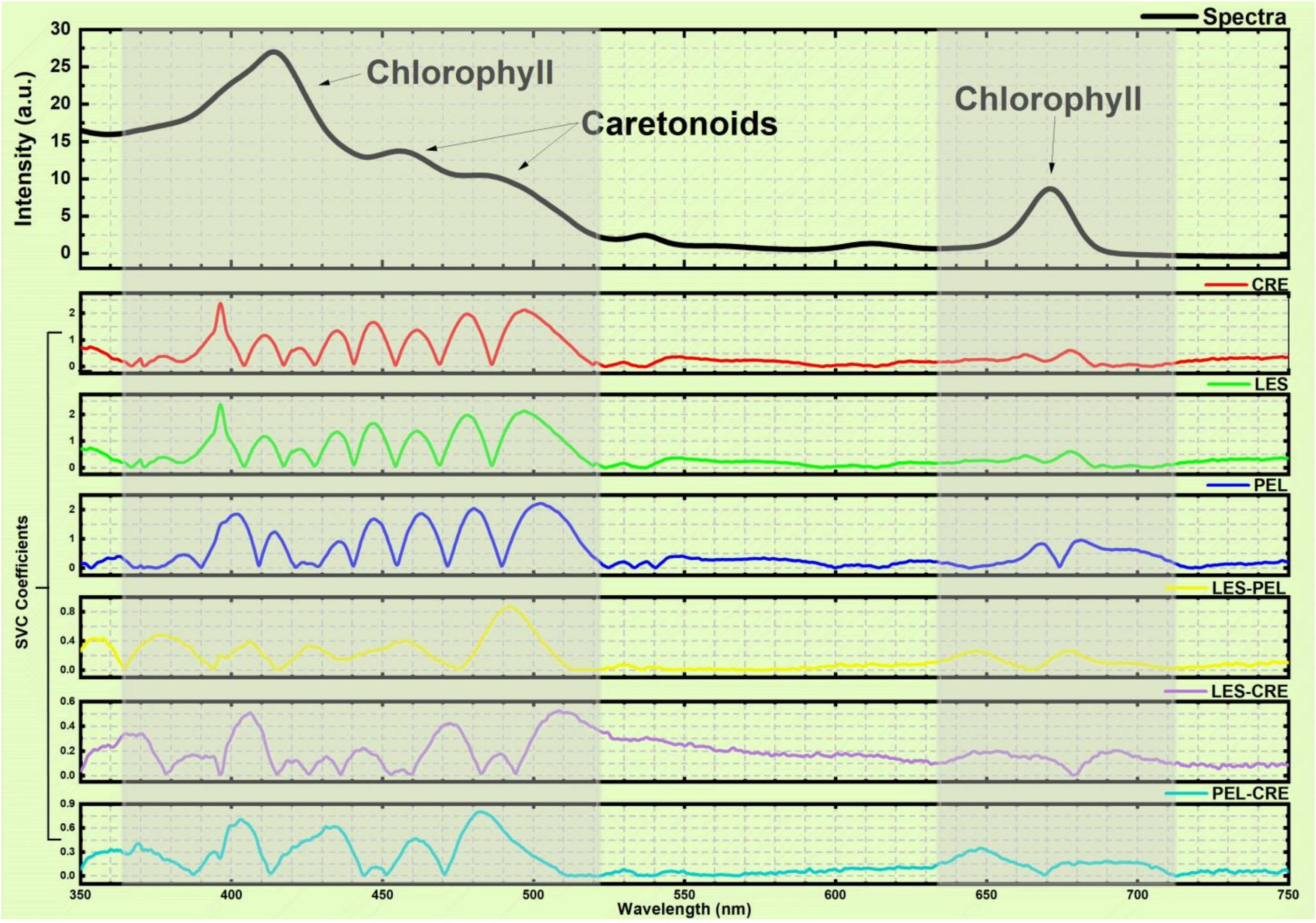

Figure S3. SVC coefficients for the absorption predictive model, in comparison with an absorption spectrum (black line). Coefficients correspond to the samples from Crete (CRE, red line), Lesvos (LES, green line) and Peloponnese (PEL, blue line), as well as the mixtures of LES-PEL (yellow line), LES-CRE (purple line) and PEL-CRE (light blue line).

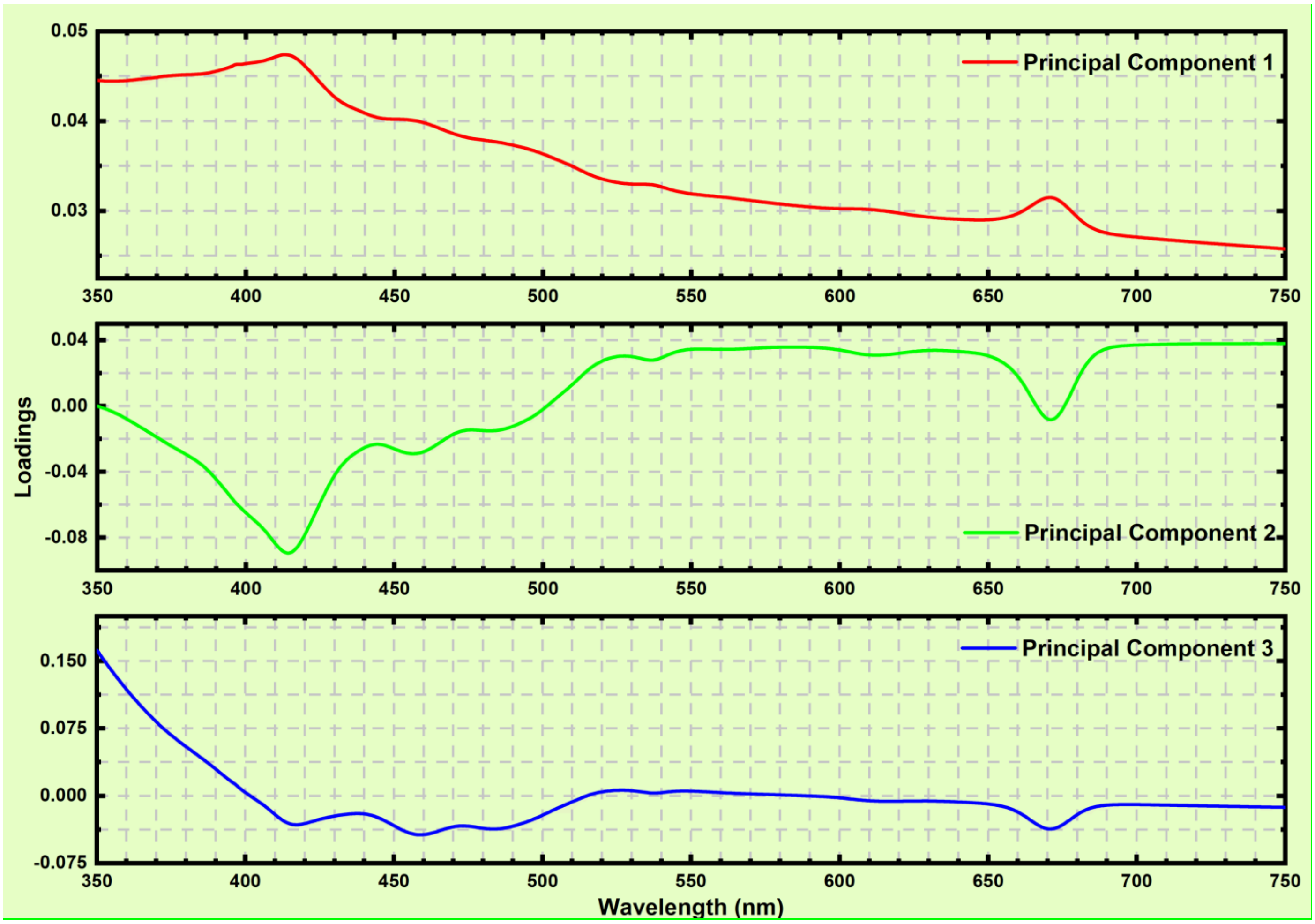

Figure S4. Loadings derived from PCA for absorption data. The red line corresponds to the first Principal Component (PC), the green corresponds to the second PC and the blue line corresponds to the third PC.

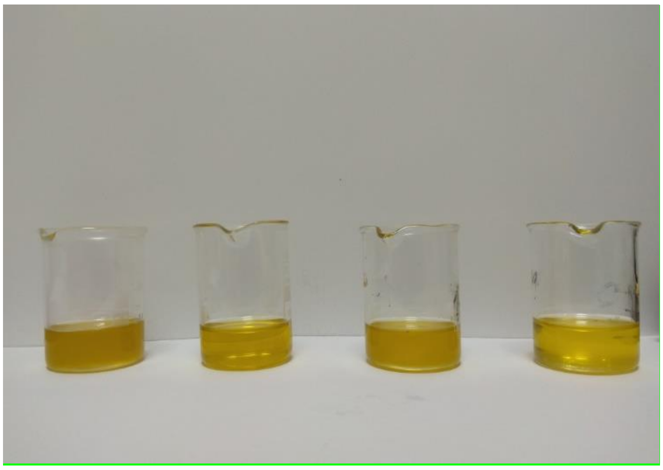

Figure S5. Samples (from left to right) from Peloponnese, Crete, Lesvos and Lesvos-Crete mixture.
