# Supplementary figures and images for: Classification of Greek Olive Oils from Different Regions by Machine Learning-Aided Laser-Induced Breakdown Spectroscopy and Absorption Spectroscopy
Source: Molecules. 2021 Feb 25;26(5):1241. doi: 10.3390/molecules26051241 (PMC7956679; doi:10.3390/molecules26051241)

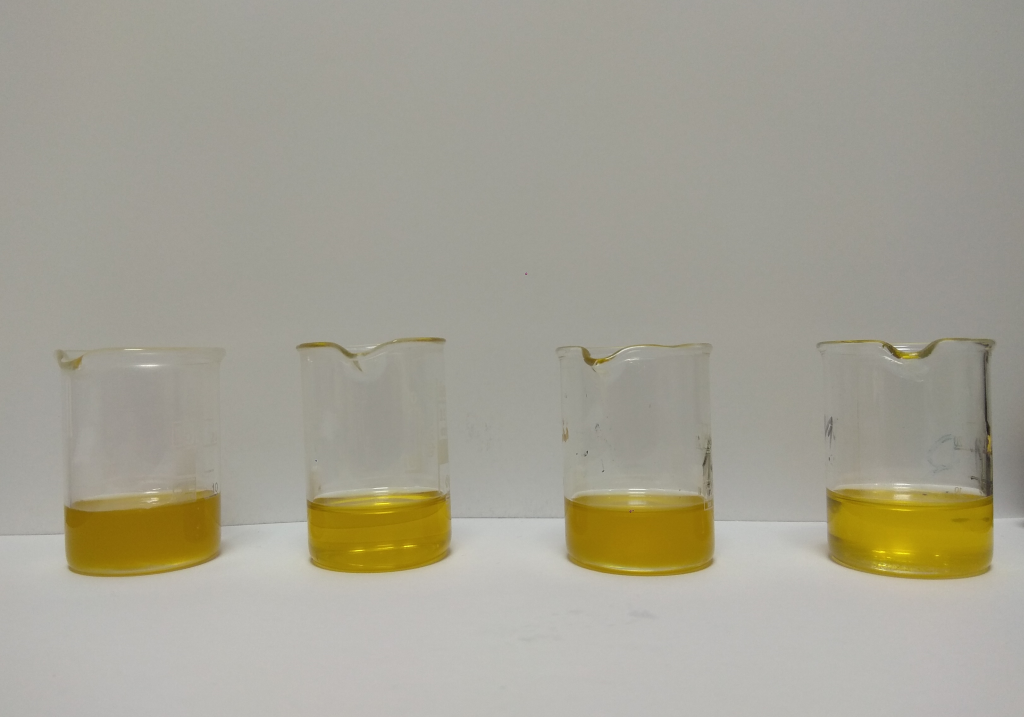

Supplement: Supplementary file 1 [file molecules-26-01241-s001.zip › molecules-1085903-supplementary/FigureS5.tif]
